# Role of Tissue-Resident Memory in Intra-Tumor Heterogeneity and Response to Immune Checkpoint Blockade

**DOI:** 10.3389/fimmu.2018.01655

**Published:** 2018-07-16

**Authors:** Kavita M. Dhodapkar

**Affiliations:** Aflac Cancer Center of Children’s Healthcare of Atlanta, Department of Pediatrics, Emory University, Atlanta, GA, United States

**Keywords:** tissue-resident memory cells, immune checkpoint blockade, tumor heterogeneity, cancer immunotherapy, immunity to cancer

## Abstract

Tissue-resident memory T (T_RM_) cells are a distinct subset of memory T cells that reside in non-lymphoid tissues for prolonged periods of time without significant recirculation providing continued immune surveillance at these sites. Recent studies suggest that T_RM_ cells are also enriched within tumor tissue. Expression of inhibitory immune checkpoints (ICPs) is particularly enriched on this subset of tumor-infiltrating T cells, suggesting that they are major targets for newer therapies targeting ICPs such as the programmed death-1 pathway. Recent studies suggest that tissue restriction of these cells without recirculation may also lead to heterogeneity of T_RM_ cells within individual metastatic lesions, ultimately leading to inter-lesional diversity. Thus, individual metastatic lesions may contain genomically distinct immune microenvironments that impact both evolution of tumors as well as the mechanisms underlying response and resistance to immune therapies. Understanding the biology of T_RM_ cells infiltrating tumors will be essential to improving immune-based approaches in diverse settings.

Immune-based approaches, particularly those based on the blockade of inhibitory immune checkpoints (ICPs) on T cells have emerged as among the most promising new strategies to treat cancer (1). An important aspect of immune therapies is their potential ability to mediate long-term control of tumors. The capacity of the immune system to mediate long-term protection, particularly against pathogens, such as in the context of vaccines, is mediated in large part by immunologic memory (2). Therefore, understanding immunologic memory mediated by T cells is likely to be important for deeper understanding of immune-mediated long-term control of tumors. It is thought that uptake of antigens from dying tumor cells by antigen-presenting cells leads to activation of anti-tumor T cells in the lymph nodes, and resultant effector memory T cells traffic back to the tumor to mediate anti-tumor effects, creating a tumor-immunity cycle (3). Several studies have shown that infiltration of primary and metastatic lesions by immune cells, particularly T cells and myeloid cells impacts outcome (4). Studies in both mice and humans suggest that there are differences in the memory T cell subsets that provide immune surveillance within lymphoid and non-lymphoid tissues (NLTs). As tumor-related mortality in most solid tumors is not due to growth of primary tumors, but rather due to the growth of metastatic tumor cells in NLTs, it is the immune surveillance in NLTs that may be critical for protective tumor immunity. In this review, we discuss newer insights into spatial aspect of immunologic memory and particularly memory T cells within NLTs in the context of tumor immunity. We will discuss emerging evidence suggesting that the biology of these tissue-resident memory (T_RM_) T cells may not only be critical for understanding and improving clinical responses to ICP blockade, but may also contribute to the complexity of immune microenvironment by creating inter-lesional heterogeneity in the setting of metastatic cancer.

## T_RM_ T Cells: Local Policemen

Initial models of T cell memory classified effector/central memory (T_EM_/T_CM_) T cells, with the effector subset implicated in surveying NLTs (5). Recent studies have identified a third subset, termed T_RM_ T cells that reside for prolonged periods in NLTs and play an important role in protective immunity (6). Mouse T_RM_ cells have been described in diverse tissues, including lung, liver, brain, as well as barrier tissues (6, 7). Murine T_RM_ cells haven been shown to mediate rapid *in situ* protection against viral, bacterial, and parasitic infections and are more effective in this regard than their circulating counterparts, including central memory T cells (7, 8). An important aspect of T_RM_-mediated immune surveillance is its regional nature. Thus in parabiotic mice that share systemic circulation, T_RM_ cells remain localized within tissues and do not cross over to equilibrate in the paired mouse carrying antigenic stimulus (6). T_RM_ cells express CD69, which is implicated in tissue retention by sequestration of the sphingosine-1-phosphate receptor (9).

Tissue-resident memory cells have also been identified in several human tissues and implicated in tissue-restricted pathology particularly in the skin, such as fixed drug eruptions (10–12). As in the mouse, human T_RM_ cells have been identified by the expression of CD69 on memory T cells within tissues, which is generally lacking in blood memory T cells (13). In humans, CD103 is expressed only in a subset of CD69+CD8+ memory T cells in some barrier tissues, but not by CD4+ memory T cells in any tissue, indicating that CD69 may be a more universal marker distinguishing both CD4+ and CD8+ memory T cells in tissues from their blood counterparts. It is notable that the proportion of T_RM_ cells differs in different tissues, with enrichment in some barrier tissues such as skin. Recent studies have also characterized transcriptional profiles of human T_RM_ cells, which resemble their murine counterparts and also illustrate that these are a distinct subset of human memory T cells (14, 15).

The pathways that regulate generation, recruitment, retention, and long-term maintenance of these T cells in NLTs remain an active area of research. New insights into transcriptional regulation of the T_RM_ differentiation are emerging and may differ between humans and mice. For example, the transcription factor Hobit/ZNF683 is exclusively expressed and required for the generation of murine T_RM_ cells after infection, but expressed at low/negligible levels on human T_RM_ cells (14, 16). In recent studies, we have shown that human and murine T_RM_ cells express NR4A1/nur77, which is also essential for T_RM_ differentiation in several murine tissues (17). Runx3 is another transcription factor that promotes the differentiation of T cells with T_RM_ phenotype (18). Retention and maintenance of T_RM_ cells may also depend on the availability of local antigen, interactions with myeloid cells as well as cytokines like TGFβ and IL-15 in NLTs (19, 20). Tissue distribution of T_RM_ cells, at least against pathogens may depend on the site of initial exposure. For example, human influenza-specific T_RM_ cells are preferentially found in the lung (21) and hepatitis-B specific T_RM_ cells particularly in the liver (22). Human bone marrow may also be a particularly interesting compartment for long-lived memory T cells with phenotype of T_RM_ cells (17, 23, 24).

## T_RM_ Cells in Tumors

Several studies have now documented that a large proportion of T cells infiltrating human tumors have T_RM_ phenotype, at least based on the expression of CD69 and CD103 (11, 12, 25–27). In some studies, these T cells were also shown to have genomic signatures consistent with those described for T_RM_ cells (11, 25, 26). This includes altered expression of genes involved in tissue retention/homing (such as downregulation of S1PR1, S1PR5, and KLF2; increase in CD69 and CD103) as well as transcription factors now functionally implicated in this phenotype (such as NR4A1, NR4A2, and Runx3) in several tissues. It is notable that some of the genes (such as Hobit) critically implicated in the biology of murine T_RM_ cells are not expressed at high levels in their human counterparts. It is notable that in mouse models of viral infections such as lymphocytic choriomeningitis virus (LCMV), T cell memory has been largely studied when the underlying viral antigen is depleted. However, the biology in human tumors or other states of persistent viral infection may differ from LCMV models and local antigen may have important implications for T_RM_ biology. Indeed, recent studies suggest that local antigen may drive proliferation of T_RM_ cells *in situ* (28, 29).

While the infiltration of tumors by T cells has in general emerged as a strong indicator of improved prognosis, the presence of T_RM_ cells within tumor-infiltrating lymphocytes (TILs) may be a particular driver of this correlation. The proportion of TILs that have T_RM_ phenotype differs between studies (for example, from 25 to 75%) and may depend in part on the nature of specific markers utilized to identify these cells as well as the specific tissue/organ studied. This subset of cells may also be enriched for tumor reactivity, which is also consistent with other studies showing enrichment of tumor reactivity such as against tumor-associated neoantigens in CD8+ memory T cells with PD1+ phenotype (26, 30). Recent studies in murine models also suggest that these cells are important contributors to protective tumor immunity (31). In this study, the presence of T_RM_ cells was modeled in the setting of autoimmune vitiligo and melanoma-specific T_RM_ cells infiltrating these lesions were shown to mediate strong tumor protection. To date, most of the data relating to the biology of T_RM_ cells in human tumor tissues are largely based on patients with solid tumors. Further studies are needed to better characterize this subset of T cells within hematologic malignancies. Below, we particularly focus on two aspects of the biology of tumor-associated T_RM_ cells, their contribution to clinical responses to ICP blockade therapies and emergence of inter-lesional heterogeneity.

## Are T_RM_ Cells a Critical Target for ICP Blockade?

Antibody-mediated blockade of inhibitory ICPs such as programmed death-1 (PD-1) have led to impressive and durable clinical regressions in several cancers (32). This is remarkable as the expression of ICPs such as PD-1 is limited to only a subset of TILs (33). The principle of ICP blockade is based on the concept of unleashing the activity of pre-existing anti-tumor T cells against the tumor (34). Studies of T cell receptor (TCR) sequencing of T cells from patients receiving anti-PD1 therapy suggests that this therapy leads to *in situ* proliferation of CD8+ T cells within tumors of patients who respond to therapy (35). The ICP expressing T cells were found to include most of the tumor reactive T cells. While such tumor-reactive T cells can be detected in peripheral blood, these cells are predominantly present within the tumor tissue. In recent studies, we and others have shown that T_RM_ cells are the dominant T cell subset expressing ICPs within the tumor microenvironment (11, 25). While most studies have described the presence of T_RM_ cells within adult tumors recent data suggest that T_RM_ cells are also enriched within pediatric tumors like glioma and are the T cell subset within these tumors that predominantly expresses ICPs (36). While T_RM_ cells were initially identified in the tumor tissue based on the expression of classic T_RM_ markers such as CD69 or CD103, gene expression studies confirmed that these T cells are a distinct subset of TILs with a genomic signature overlapping with T_RM_ signature. Importantly, although CD69 is well studied as a T cell activation marker, the genomic profiles of CD69+ T_RM_ cells are distinct from activated T cells and instead enriched for tissue retention genes (25). Therefore, while tumor tissue contains antigens recognized by these cells, and T_RM_ cells express CD45RO consistent with memory T cells, they are genomically distinct from simply activated effector memory T cells. Recent studies in murine tumor models also support the importance of tumor-infiltrating T_RM_ cells in mediating long-term control of melanoma tumors (31). The relationships between T_RM_ cells and other populations such as stem memory cells implicated as targets of proliferative burst after PD-1 blockade need further study (37). Further studies are also needed to better characterize the proportion of tumor infiltrating T_RM_ cells that are truly tumor specific.

The concept that T_RM_ cells may be major targets of ICP blockade therapies is consistent with emerging insights into their functional properties. T_RM_ cells seem to provide a dual role that encompasses both protection and regulation. Thus, while human T_RM_ cells in NLTs can produce higher levels of effector cytokines, such as IFNγ, IL2, and TNF, they also produce higher levels of immune regulatory cytokines such as IL10 (14, 15). Moreover, T_RM_ cells also express higher levels of ICPs, such as CTLA4, PD-1, TIM-3, and LAG-3 (14, 25). T_RM_ cells also seem to have a quiescent phenotype, which may be essential for their ability to survive long-term in tissues, being poised for activation but not harming tissues (17). Antibody-mediated blockade of ICPs such as PD-1, therefore, provides a mechanism for activation of these T cells *in situ*. The precise nature of the activation signal may differ between CTLA4 and PD-1 blockade (or combination thereof) (38).

The concept that T_RM_ cells within tumors may be major targets of ICP blockade has several implications for immune therapies. Vaccines that foster the generation of T_RM_ cells may be best suited for combination with ICP blockade (39). The ability of T_RM_ cells to mediate long-term residence in tissues may help to explain why clinical responses to ICP blockade have been durable. Along these lines, strategies that help to maintain or even enrich these T_RM_ pools may allow enhanced durability of responses. It would also be important to better understand the nature of antigenic targets on tumors recognized by these T cells, and the impact of tumor genetics as well as other cells in the tumor microenvironment on the functional properties and retention of these cells.

## Do T_RM_ Cells Contribute to Intra-Tumor Heterogeneity of Tumors?

Advances in cancer genomics and particularly the capacity to sequence multiple lesions in the same patient or even different parts of the same tumor have demonstrated a complex and heterogeneous landscape with varying sub-clonal architecture; studies have also suggested a potential impact of such intra-tumoral heterogeneity on clinical outcome (40, 41). However, the degree to which the genetics of the microenvironment contributes to intra-tumoral heterogeneity is less clear. Diversity within the immune microenvironment may in principle not only impact the mechanisms underlying response or resistance to immune therapies but also evolution of tumors in individual metastases. Advances in TCR sequencing provide an opportunity to gain some insights into the nature and genetics of T cells infiltrating tumor lesions. While the same antigenic epitope may in principle be recognized by different TCRs, they are likely to differ in terms of their affinity or functional properties.

**Figure 1:**
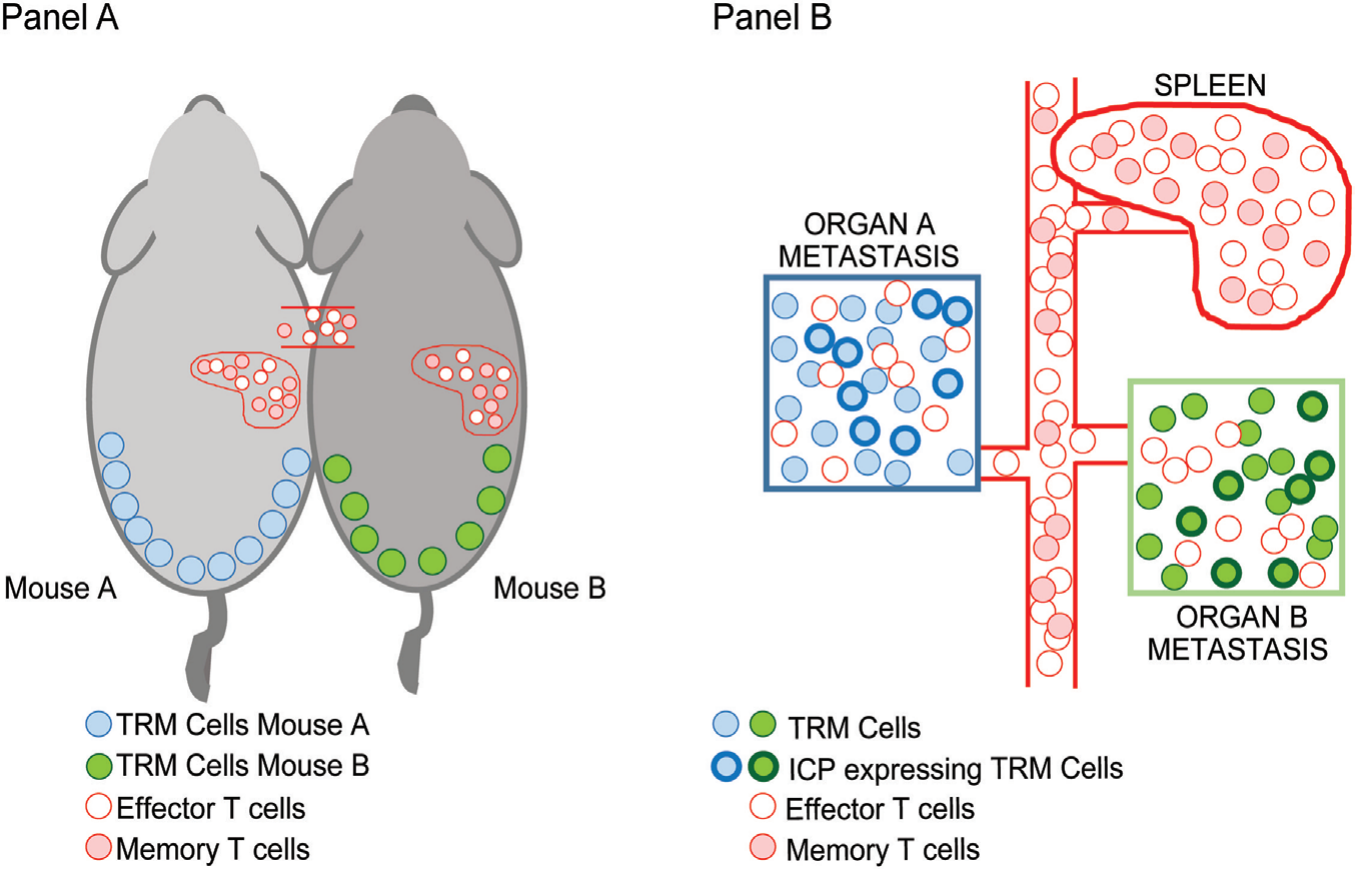
Inter-lesional heterogeneity in metastatic cancer and biology of tissue-resident memory (T_RM_) cells. T_RM_ T cells were identified in mice based on their restriction to non-lymphoid tissues and lack of recirculation. This was demonstrated using parabiotic mice **(A)** that share the same systemic circulation. Figure shows that T_RM_ cells in the skin (blue/green) do not equilibrate between mice, while other effector/memory T cells (pink/red) do. In the setting of advanced cancer in humans **(B)**, individual metastatic lesions can be observed in diverse tissues that share systemic circulation analogous to the parabiotic mice. Sequencing of T cell receptors (TCRs) in individual lesions from the same patient demonstrated that dominant TCRs in each of the lesions were non-overlapping and that the inter-lesional heterogeneity of TCRs exceeded differences in neoantigens. Importantly, T_RM_ cells were the major contributors to this heterogeneity suggesting that they do not equilibrate between lesions as in parabiotic mice in Ref. (25). A subset of T_RM_ cells that infiltrate these tumors express inhibitory immune checkpoints such as PD1 (shown by bolded outlines).

In the setting of advanced or metastatic cancer, tumor cells grow as discrete lesions in diverse NLTs. These lesions by definition share the systemic circulation of the host and could in principle be likened to the situation in parabiotic mice that share systemic circulation. As discussed earlier, a characteristic feature of T_RM_ cells is tissue residence without recirculation, revealed by lack of equilibration in parabiotic mice. We hypothesized that if T_RM_ cells within individual tissues (e.g., lung or liver or skin lesions) indeed remain local, then dominant TCRs within individual metastatic lesions in the same patient would not equilibrate even if the oncogenic mutations or neoantigen-load were largely shared between these lesions (Figure 1). Concurrent sequencing of tumor cells as well as TCRs from individual lesions in patients with advanced melanoma supported this hypothesis; as expected, the inter-lesional diversity of TCRs was mostly accounted for by TCRs from T_RM_ subset of TILs (25). Differences in dominant TCRs between individual lesions from the same patient is consistent with lack of equilibration of TCRs between individual metastatic lesions even though they may share a major component of neoantigen load. However, the mechanisms that limit this equilibration need to be better defined; our current hypothesis is that it may relate to the lack of recirculation of tissue-resident TCRs, or their relative tissue retention, both consistent with T_RM_ biology.

The concept that T_RM_ cells infiltrating tumor tissues may exhibit local residence and little recirculation has several implications for immune therapies, immune monitoring, and cancer biology. If the individual metastatic lesions are established early, and carry different TCRs, then the level of immune pressure in individual lesions may differ and provide a pathway for divergent genomic evolution (42). Along the same lines, it may be important to carefully consider the specific site of tissue biopsy when evaluating the results of immune monitoring. It should be noted, however, that the impact of ICP blockade on T_RM_ homeostasis and redistribution *in vivo* in humans remains understudied and may add additional layers of complexity. Studies harvesting TILs for adoptive transfer are now entering the clinic in diverse cancers. If the dominant TCRs differ between individual lesions, it may be desirable to harvest and pool T cells from more than one lesion to optimize efficacy of such cell therapies. Finally, if the T cells in individual lesions differ, then it raises the potential that multiple mechanisms of immune resistance may be simultaneously operative in the same patient (43); along these lines, isolated progression at a single site in the face of continued regression at other sites may not reflect systemic loss of tumor control in the context of immune therapies. Clinicians have already come to appreciate this difference between immune therapies as compared to chemotherapies and often utilize localized therapies to tackle such lesions.

## Summary

In summary, T_RM_ cells within tumor lesions are likely to gain increasing importance as targets of immune therapies as well as deeper understanding of cancer biology and evolution. It is likely that optimal integration of these immune therapies will require attention to the unique biology of these immune cells and exploit their regional nature of enhance tumor immunity with reduced systemic toxicity.

## Author Contributions

The author confirms being the sole contributor of this work and approved it for publication.

## Conflict of Interest Statement

The author declares that the research was conducted in the absence of any commercial or financial relationships that could be construed as a potential conflict of interest.
